# Activation of host transient receptor potential (TRP) channels by praziquantel stereoisomers

**DOI:** 10.1371/journal.pntd.0006420

**Published:** 2018-04-18

**Authors:** Gihan S. Gunaratne, Nawal A. Yahya, Peter I. Dosa, Jonathan S. Marchant

**Affiliations:** 1 Department of Pharmacology, University of Minnesota, Minneapolis, United States of America; 2 Institute for Therapeutics Discovery and Development, University of Minnesota, Minneapolis, United States of America; 3 Department of Cell Biology, Neurobiology and Anatomy, Medical College of Wisconsin, Milwaukee, United States of America; McGill University, CANADA

## Abstract

The anthelmintic praziquantel (±PZQ) serves as a highly effective antischistosomal therapy. ±PZQ causes a rapid paralysis of adult schistosome worms and deleterious effects on the worm tegument. In addition to these activities against the parasite, ±PZQ also modulates host vascular tone in blood vessels where the adult worms reside. In resting mesenteric arteries ±PZQ causes a constriction of basal tone, an effect mediated by (*R*)-PZQ activation of endogenous serotoninergic G protein coupled receptors (GPCRs). Here, we demonstrate a novel vasodilatory action of ±PZQ in mesenteric vessels that are precontracted by high potassium-evoked depolarization, an effect previously reported to be associated with agonists of the transient receptor potential melastatin 8 channel (TRPM8). Pharmacological profiling a panel of 17 human TRPs demonstrated ±PZQ activity against a subset of human TRP channels. Several host TRP channels (hTRPA1, hTRPC3, hTRPC7) were activated by both (R)-PZQ and (S)-PZQ over a micromolar range whereas hTRPM8 showed stereoselective activation by (*S*)-PZQ. The relaxant effect of ±PZQ in mesenteric arteries was caused by (*S*)-PZQ, and mimicked by TRPM8 agonists. However, persistence of both (S)-PZQ and TRPM8 agonist evoked vessel relaxation in TRPM8 knockout tissue suggested that canonical TRPM8 does not mediate this (S)-PZQ effect. We conclude that (S)-PZQ is vasoactive over the micromolar range in mesenteric arteries although the molecular mediators of this effect remain to be identified. These data expand our knowledge of the polypharmacology and host vascular efficacy of this clinically important anthelmintic.

## Author summary

Praziquantel is a key drug for combating diseases caused by parasitic flatworms. It is the therapeutic mainstay for treatment of schistosomiasis, a disease that afflicts over 200 million people worldwide. In this study, we investigate potential molecular targets of praziquantel, and demonstrate interactions with several members of the transient receptor potential (TRP) ion channel family over the micromolar range. These interactions with endogenous host TRP channels may contribute to regulation of vascular contractility in the blood vessels where the mature parasites reside.

## Introduction

Schistosomiasis is a socioeconomically devastating helminth infection afflicting over 200 million people worldwide [1]. The resulting disease burden of chronic schistosomiasis is estimated to encumber third world economies with an annual loss of 70 million disability-adjusted life years [2, 3]. In infected individuals, the prolific egg laying capacity of paired adult worms (>1000 eggs/day deposited in tissues, [4]) triggers localized inflammatory responses around eggs trapped within host tissues. Chronic infections progress toward fibrosis and obstructive disease in gastrointestinal tissues and liver (*S*. *mansoni*, *S*. *japonicum*), genitourinary disease (*S*. *haematobium*), anemia, undernutrition and a heightened risk for other comorbidities. Effective drug therapy for schistosomiasis is therefore a healthcare priority [1–3].

The drug praziquantel (±PZQ) has served as the stalwart antischistosomal therapy since the 1980s and the need for ±PZQ is significant [5]. Thankfully, the drug has remained effective over three decades of clinical use, although there are certainly features of ±PZQ that are less than optimal. These include anxiety over the emergence of drug resistance in face of selective pressures imposed by mass distribution efforts, a refractoriness of juvenile worms to PZQ, our lack of understanding over the molecular target(s) of PZQ and an inability to improve on PZQ by chemical derivatization of the drug [6, 7]. Certainly, a better understanding of how ±PZQ works would catalyze future drug development efforts toward the next generation of antischistosomal compounds.

Addition of ±PZQ to adult schistosomes causes an acute Ca^2+^ influx, rapid paralysis of the musculature and a more chronic tegumental damage that aids immunological elimination of worms from the host. Efficacy *in vitro* and *in vivo* is associated with the action of (*R*)-PZQ as the more active enantiomer (eutomer) in the clinical formulation [8, 9], underpinning effort to develop an enantiopure clinical formulation [10]. ±PZQ also displays activity against target(s) in the host [11, 12], including vasoconstriction of the mesenteric blood vessels inhabited by the adults worms, an effect caused by (*R*)-PZQ stimulation of endogenous serotoninergic GPCRs [13]. The distomer (S)-PZQ also exhibits host bioactivity: it is associated with an unpleasant bitter taste effect [14] and effects a transient translocation (‘hepatic shift’) of *S*. *mansoni* worms from the splanchnic beds to the liver on administration [9] despite the appreciated lack of efficacy of (S)-PZQ against worms *in vitro*. Recent work has revealed activity of ±PZQ against the human transient receptor potential melastatin 8 channel (TRPM8, [15]), although the efficacy of the individual enantiomers at regulating TRPM8 are undefined. TRP channels belong to a superfamily of ion channels that respond to a broad diversity of stimuli and chemotypes underpinning many elements of our sensory physiology [16, 17]. Schistosome TRPs are themselves promising targets for their druggability [18, 19]. Collectively, both recent reports underscore considerable progress in defining activities and target(s) of ±PZQ action in the human host [13, 15].

Here, we report a novel vasodilatory action of (S)-PZQ in contracted mesenteric vessels. Based on previously published data implicating TRPM8 channels in this vasodilatory effect in rat mesenteric arteries [20], further prioritized by the work of Babes et al. [15] showing activation of TRPM8 by ±PZQ, activity of ±PZQ on endogenous TRPs that regulate myogenic tone was suspected. This study was designed to investigate the interaction of (*R*)-PZQ and (*S*)-PZQ with human TRPs, and test the possibility that such interactions regulate mesenteric vessel tone.

## Methods

### Reagents

±PZQ was purchased from Sigma and individual enantiomers–(*R*)-PZQ and (*S*)-PZQ–were resolved following protocols published by Woelfle *et al*. [10]. Icilin and WS-12 were from R&D Systems and all other ligands were sourced from Sigma-Aldrich. HEK-293 cell lines were sourced from ATCC (CRL-1573) and found to be negative for mycoplasma contamination. Cell culture reagents were from Invitrogen.

### Molecular cloning

Human TRPM8 cDNA was a VersaClone from R&D Systems (RDC0188). Plasmids encoding human TRPA1 and human TRPV1 cDNA were purchased from DNASU plasmid repository (HsCD00080227 and HsCD00081472, respectively). The TRP channel coding sequences were subcloned into pCS2+ to introduce a COOH-term myc tag using the InFusion HD method (Clontech), HindIII/XhoI restriction enzymes (NEB) and the following primers: TRPM8 F–TGGGGACGTCGGAGC-*aagctt*-gccaccatgtcctttagagcag; TRPM8 R–AAATCGATGGGATGC-*ctcgag*-tttgattttattagcaatctctttcagaagacc; TRPA1 F-GGACGTCGGAGC-*aagctt*- atgaagcgcagcctgagg; TRPA1 R-TCGATGGGATGC-*ctcgag*-aggctcaagatggtgtgtttttgc; TRPV1 F–GGACGTCGGAGC-*aagctt*-atgaagaaatggagcagcacag; TRPV1 R-TCGATGGGATGC-*ctcgag*-cttctccccggaagcgg (where upper case specifies vector-specific sequences, italics indicate restriction sites, and lower case indicates TRP channel specific sequences). Primers are listed in a 5’ to 3’ orientation.

### Measurements of vascular tone

Swiss Webster mice (female, 10–13 weeks) were sourced from Charles River Laboratories. Measurements of mouse mesenteric vessel tone were made using wire myography using a four channel myograph system (DMT, Aarhus, Denmark). Vessel strips isolated from second order mesenteries were equilibrated for ≥30 min in gassed (95% O_2_, 5% CO_2_), physiological saline solution (PSS, 130mM NaCl, 4.7mM KCl, 1.18mM KH_2_PO_4_, 1.17mM MgSO_4_, 14.9mM NaHCO_3_, 5.5mM dextrose, 0.026mM EDTA, 1.6mM CaCl_2_, pH 7.4 at 37°C). To identify the optimal pre-stretch value for experiments, a normalization factor (IC_1_/IC_100_) was calculated for individual test strips [21, 22], defined as the ratio of the internal circumference at which the maximum response to vasoconstriction (KCl, plus 40μM norepinephrine) was observed (IC_1_), divided by the internal circumference at which a transmural wall pressure of 100mm of Hg is attained on a length-tension plot overlayed with a La Place transformation isobar (IC_100_). After vessel equilibration, reactivity was measured under isometric conditions in response to KCl (KPSS, 74.7mM NaCl, 60mM KCl, 1.18mM KH_2_PO_4_, 1.17mM MgSO_4_, 14.9mM NaHCO_3_, 5.5mM dextrose, 0.026mM EDTA, 1.6mM CaCl_2_, pH 7.4 at 37°C) or indicated ligands as detailed for individual experiments. Homozygous TRPM8 knockout (KO) mice, harboring a premature truncation within the cytoplasmic NH_2_-terminal domain of TRPM8 [23], were sourced from the Jackson Laboratory (Trpm8^tm1Jul^/Trpm8^tm1Jul^, female, 16–18 weeks). For these experiments, CR7BLBL/6J mice were used as age and strain matched controls.

### TRP channel profiling

±PZQ, (*R*)-PZQ and (*S*)-PZQ were screened against a panel of 17 human TRP channels (SB Drug Discovery, Glasgow). For all hTRPs, except for TRPM5, individual channel constructs were stably expressed in HEK cell lines. TRMP5 was expressed in a stable CHO cell line. In preparation for the assays, cells were trypsinized, counted and seeded (50,000 cells/well) in black, clear-bottomed 96 well plates and incubated overnight. The following day, cells were loaded with a fluorescent indicator (FLIPR Calcium 5 Assay kit for TRPA1, TRPV1, TRPV2, TRPV3, TRPV4, TRPV5, TRPC1, TRPM2, TRPM3 and TRPM8, or a membrane potential dye (FLIPR Membrane Potential Red Assay Kit for TRPC3, TRPC4, TRPC5, TRPC6, TRPC7, TRPM4 and TRPM5) prepared according to the manufacturer’s instructions in HEPES buffered Hank’s balanced salt solution (HBSS). Dye solution (10μl) was added to appropriate wells and incubated at 37°C for 1 hour. All assays were performed at room temperature. Compounds were tested at 0.3, 1, 3, 10, 30, 100μM and 300μM in triplicate in both agonist and antagonist mode to determine EC_50_ and IC_50_ values, which were compared with reference compounds. Compounds were screened at a final DMSO concentration of 0.5%. Plates were screened using a Flexstation (Molecular Devices, FX01138), monitoring fluorescence values every ~1.52 seconds. For ‘agonist mode’ testing, 10μl of the appropriate test compound, or standard agonist, was added after 20 seconds and fluorescence monitored for 2 minutes at λ_ex_ = 485nm, λ_em_ = 525nm for Ca^2+^ imaging and λ_ex_ = 530nm, λ_em_ = 565nm for membrane potential measurements. For ‘antagonist mode’ testing, test compounds and standard inhibitors were added to appropriate wells and incubated for 10 minutes at room temperature prior to addition of standard agonist compound.

### Confocal Ca^2+^ imaging in mammalian cell lines

HEK293 cells (ATCC CRL-1573.3) were cultured in DMEM supplemented with 10% fetal bovine serum (FBS), penicillin (100 units/ml), streptomycin (100 μg/ml), and L-glutamine (290 μg/ml). Cells were transiently transfected (Lipofectamine LTX, Thermo Fisher) at a density of 2x10^6^ cells per T-25 cell-culture flask with TRP channel cDNA. For Ca^2+^ imaging assays, HEK293 cells were seeded onto 8-chambered coverglass slides (Thermo Fisher, 115411PK), at a density of 1x10^4^ cells one day prior to imaging. Cells were washed twice with HBSS, and incubated with fluo-4-AM (4μM), pluronic F127 (0.4%) and probenecid (2.5mM) for 25 minutes at room temperature. Cells were then washed twice with HBSS, and left at room temperature (30 minutes) for de-esterification. Dishes were mounted on an Olympus IX81 microscope and fluorescence changes (λ_ex_ = 488nM, λ_em_ = 513±15nm bandpass filter) monitored using a Yogokawa spinning disk confocal (CSU-X-M1N) and an Andor iXon Ultra 888 EMCCD camera.

### Ethics statement

Tissue harvesting followed ethical regulations approved by the University of Minnesota IACUC committee (Protocol #1606–33903). Animal husbandry procedures followed requirements outlines in the Public Health Service Policy on Humane Care and Use of Laboratory Animals and the Animal Welfare Act.

## Results

**Fig 1:**
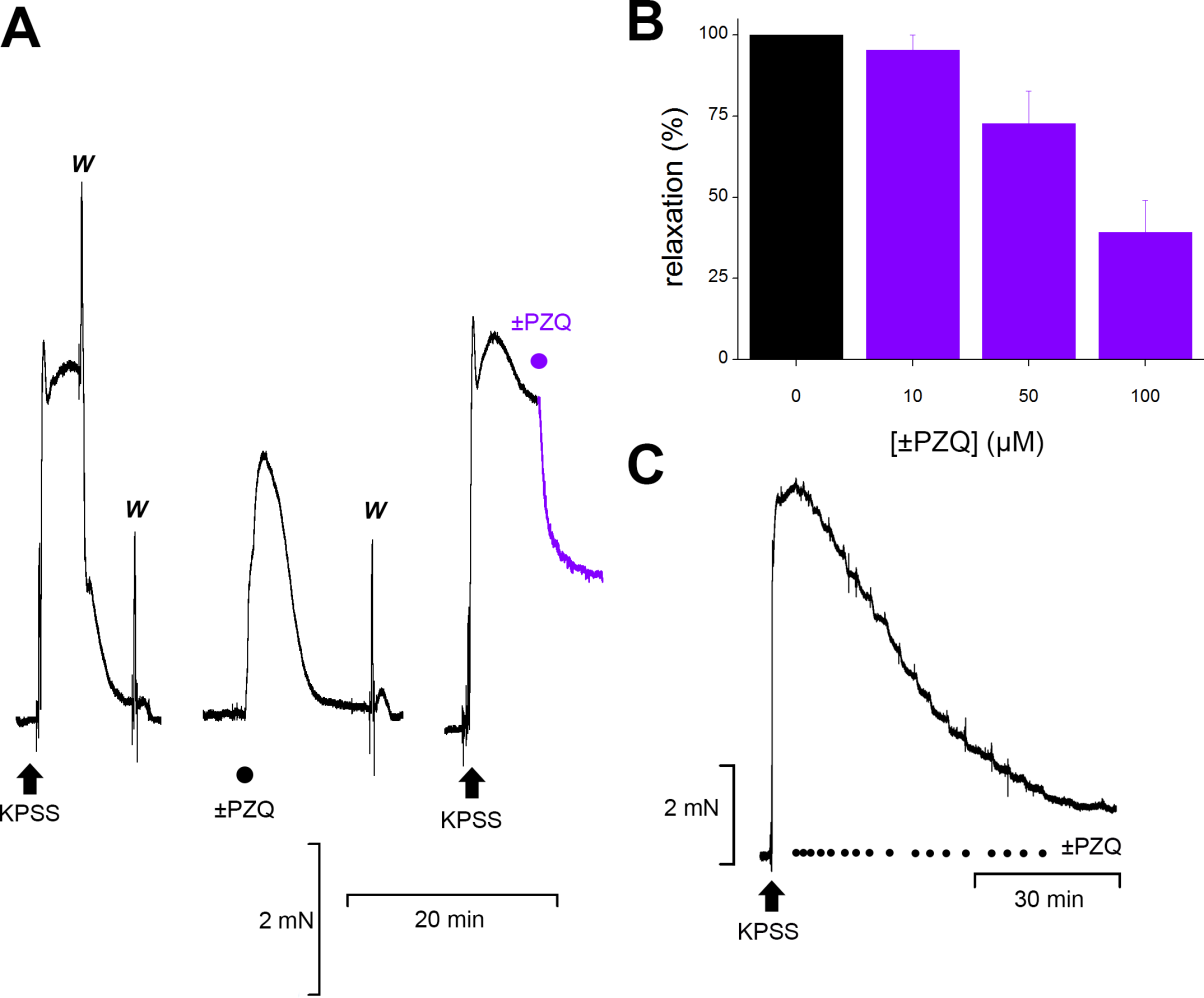
±PZQ causes relaxation of precontracted mesenteric arteries. (**A**) Changes in tension measured in mouse mesenteric artery vessel strips evoked by KPSS (left, arrow), ±PZQ (circle, 100μM) added at basal tone (middle) and KPSS (arrow) followed by addition of ±PZQ to a contracted vessel (purple circle and trace, right). Solution exchanges to physiological saline shown as ‘w’ (wash). (**B**) Dose-response relationship quantifying peak relaxation evoked by indicated concentrations of ±PZQ. (**C**) Effect of repeated additions of ±PZQ (10μM, black circles) without solution exchange on contractile tone in a vessel contracted by solution exchange to KPSS (arrow).

The contractile tone of vessel strips isolated from mouse mesenteric arteries was evaluated using wire myography. A typical experiment trace is shown in Fig 1, where mounted vessel strips exhibited a sustained contraction to high K^+^ media (KPSS) that rapidly reversed upon solution exchange (Fig 1A). At resting tone, addition of ±PZQ caused a marked contraction, consistent with recent data showing vasoconstriction mediated by (*R*)-PZQ activation of host 5-HT_2B_ receptors (Fig 1A, [13]). However, an additional action of ±PZQ was observed in vessels precontracted by KPSS exposure. Addition of ±PZQ to vessels contracted with KPSS caused a marked relaxation (Fig 1A). This vasodilatory effect of ±PZQ was dose-dependent, and sufficient to relax the contracted vessel by 61±9% at high concentrations of ±PZQ (100μM, Fig 1B). Relaxation evoked by ±PZQ was phasic, with successive additions of ±PZQ (10μM) resulting in a dose-dependent relaxation of vessel tone toward precontracted levels (Fig 1C).

**Fig 2:**
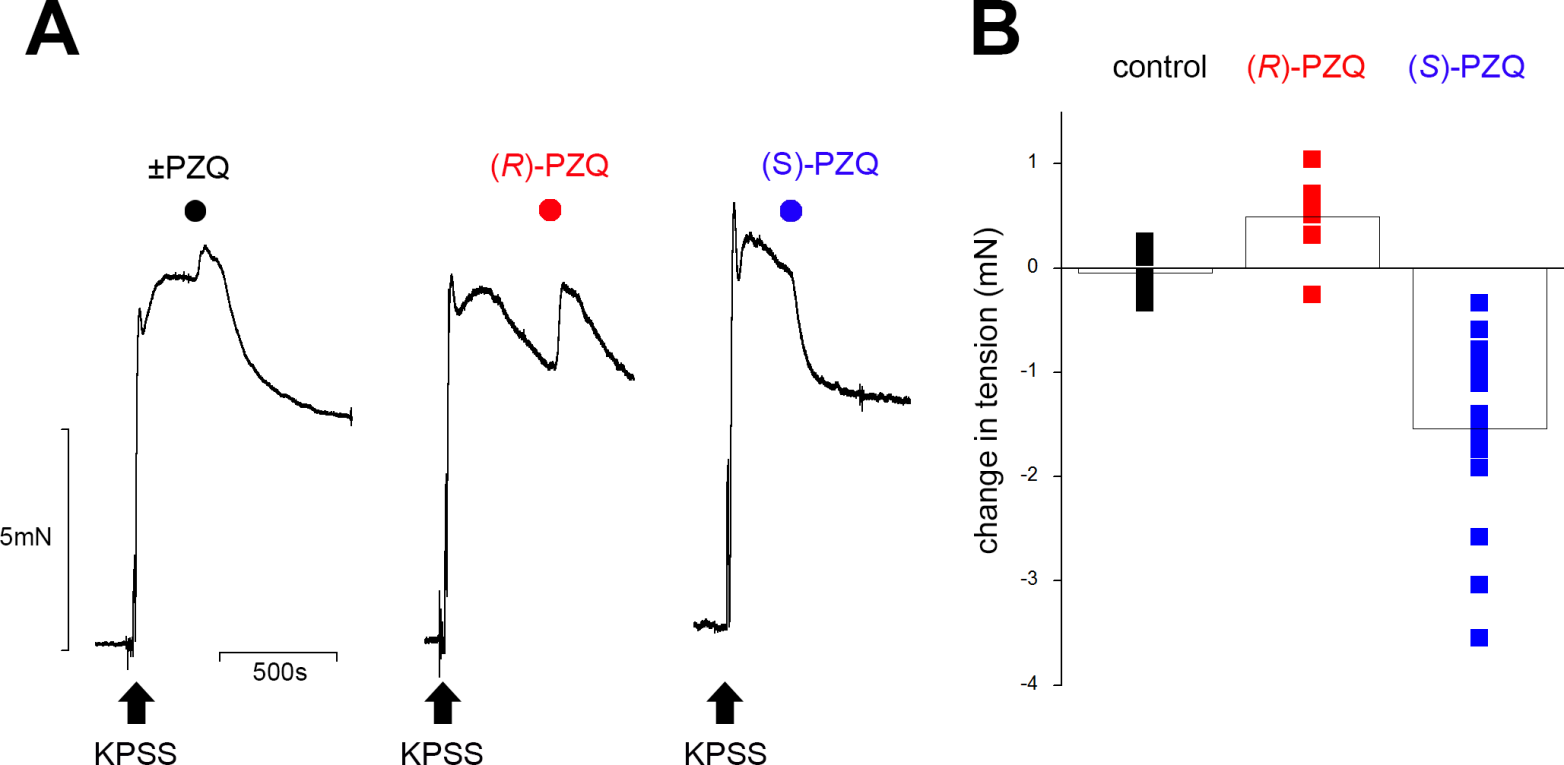
(*S*)-PZQ decrease tone of contracted mesenteric arteries. (**A**) Representative tension recordings showing addition of ±PZQ (left), (*R*)-PZQ (50μM, middle) and (*S*)-PZQ (50μM, right) during a KPSS-evoked contraction. (**B**) Individual data points (squares) show magnitude of tension change measured at a fixed time interval (1 min) after addition of (*R*)-PZQ (red) or (*S*)-PZQ (blue) relative to control traces (DMSO, vehicle) during a KPSS-evoked contraction. Each data point represents a single measurement from a unique vessel (n≥6 measurements for each condition). Average of all measurements represented by the bar chart.

The ability of the separated enantiomers, (*R*)-PZQ and (*S*)-PZQ to cause this partial vasodilation of KPSS-precontracted vessels was examined (Fig 2). The decrease in tension evoked by ±PZQ was mimicked by addition of (*S*)-PZQ (Fig 2A). In contrast, bath application of (*R*)-PZQ was associated with an initial, small contraction possibly reflecting residual serotonergic tone (Fig 2A). To quantify these effects, measurements of changes in tension 1 minute after addition of (*S*)-PZQ or (*R*)-PZQ to capture these initial changes in myogenic tone. These data confirmed that the vasodilatory action of ±PZQ on precontracted mesenteric artery strips was predominantly mediated by (*S*)-PZQ (Fig 2B).

**Fig 3:**
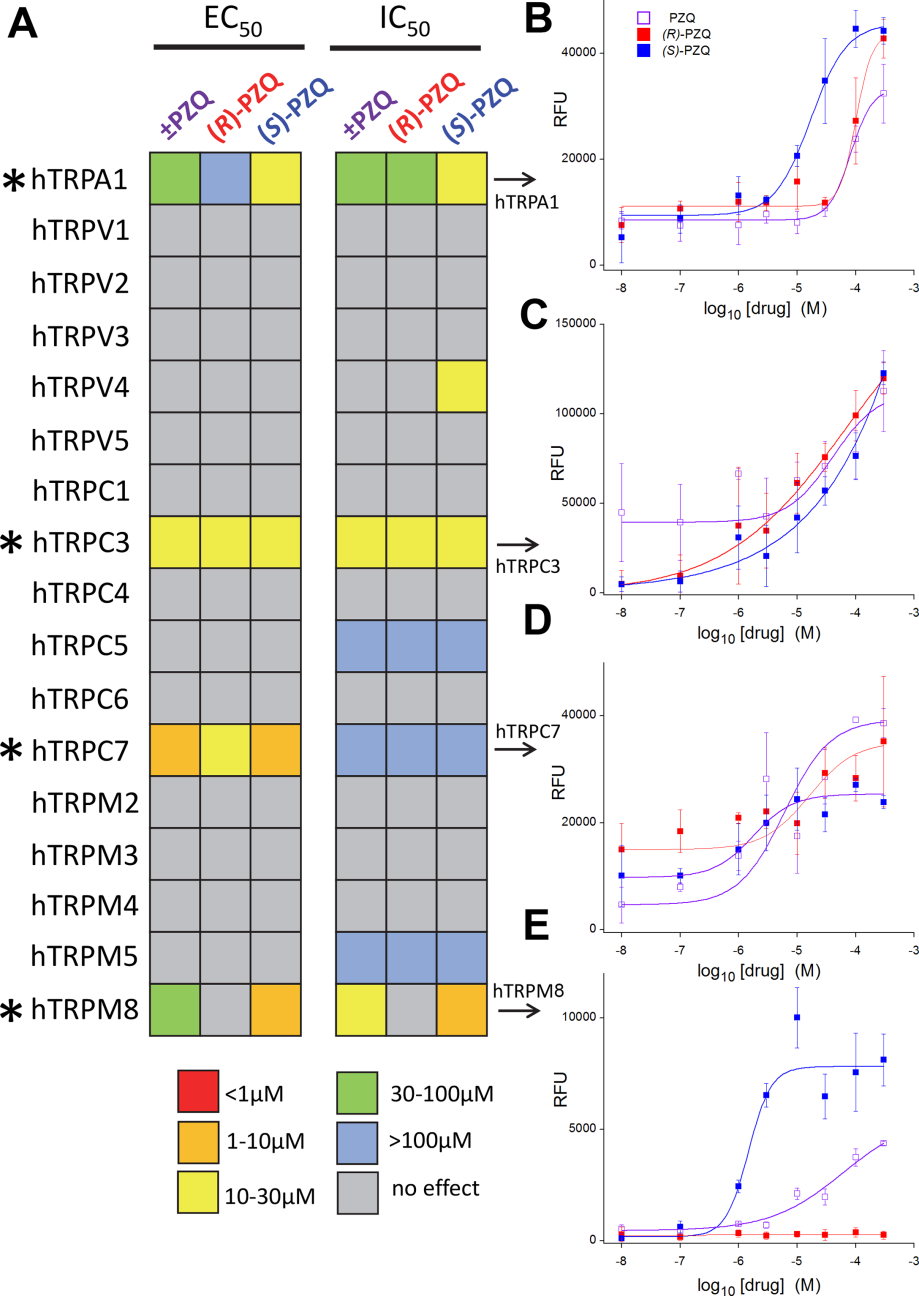
Interrogating a TRP channel panel for praziquantel activity. (**A**) Graphical schematic of results from primary screen measuring activation (EC_50_, left) or inhibition (IC_50_, right) of 17 individual human TRP channels to ±PZQ, (*R*)-PZQ and (*S*)-PZQ. EC_50_ and IC_50_ values are color encoded as per legend key, where increasing warm coloration represents higher resolved potency in assays. Responsive hTRPs are identified with asterisks (*). (**B-E**) Individual dose-response curves for activation of (B) hTRPA1, (C) hTRPC3, (D) hTRPC7 and (E) hTRPM8 by ±PZQ (open purple squares), (*R*)-PZQ (red squares) and (*S*)-PZQ (blue squares).

Phasic vasorelaxation of mouse mesenteric arteries has previously been associated with the action of agonists of the transient receptor potential melastatin 8 channel (TRPM8, [24, 25]) under a similar contractile paradigm. This observation has especial relevance given recent data showing that ±PZQ activates TRPM8 in both heterologous expression experiments, as well as in assays for endogenous TRP activity in dorsal root ganglion neurons [15]. These observations merited profiling of ±PZQ action against a broad panel of human TRP channels (hTRPs), including TRPM8. Therefore, a primary screen was performed against stable cell lines expressing individual hTRPs, using either a Ca^2+^-sensitive fluorescent dye, or a membrane-potential reporter as a readout for channel activity. Responses to ±PZQ, (*R*)-PZQ and (*S*)-PZQ were measured in triplicate in both ‘agonist-mode’ (addition of ±PZQ, (*R*)-PZQ or (*S*)-PZQ) and ‘antagonist-mode’ (inhibition of response to a channel activator by either ±PZQ, (*R*)-PZQ and (*S*)-PZQ). If functional effects were resolved, EC_50_ (‘agonist-mode’) or IC_50_ (‘antagonist-mode’) values were determined and represented as a heat-map for ease of comparison (Fig 3A). Several conclusions can be drawn from this primary screening dataset. First, ±PZQ displayed activity against only a subset of screened hTRPs—hTRPA1, hTRPC3, hTRPC7 and hTRPM8. Second, these effects occurred over the micromolar range. Third, these effects were predominantly attributable to (*S*)-PZQ activity as the more active enantiomer, or—in the case of TRPM8 - (*S*)-PZQ as the sole active enantiomer. Finally, the ability of PZQ enantiomers to both stimulate and inhibit hTRP activity implied action as partial agonists. Individual dose response curves for hTRPA1, hTRPC3, hTRPC7 and hTRPM8 activation by each ligand are shown (Fig 3B–3E).

**Fig 4:**
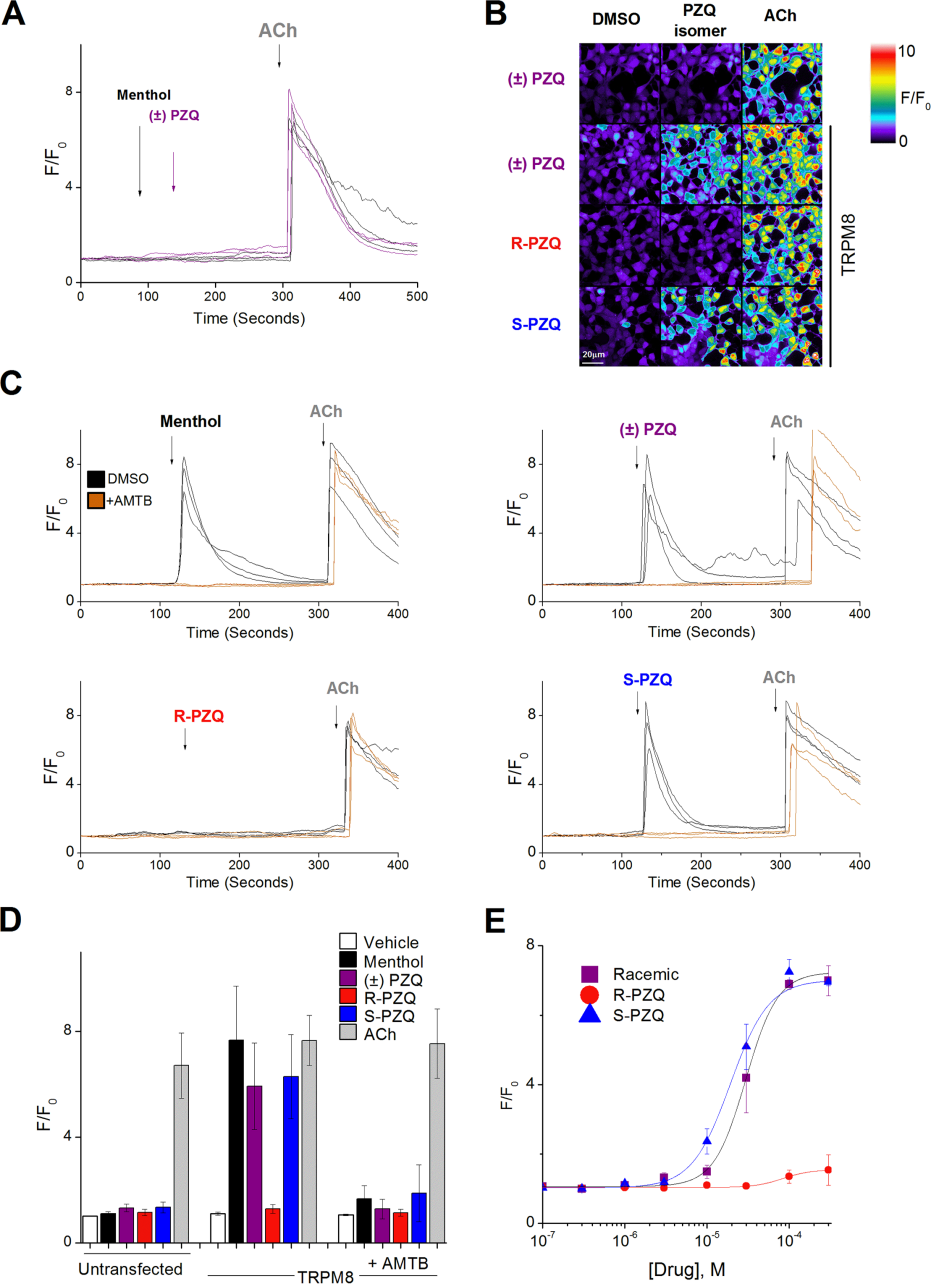
Stereoselective activation of hTRPM8 by (*S*)-PZQ. (**A**) Representative fluorescence traces from cells loaded with fluo-4-AM in a HEK293 cell line following addition of menthol (300μM), or ±PZQ (100μM) followed by ACh (100μM). (**B**) Pseudocolored confocal images from the field of view are displayed following addition of vehicle (DMSO, 0.05%), (*R*)-PZQ or (*S*)-PZQ (50μM), and ACh (100μM) in untransfected HEK293 cells (top) or cells transfected with hTRPM8 (bottom three panels). (**C**) Representative fluorescence traces from cells loaded with fluo-4-AM in a HEK293 cell line transfected with hTRPM8 following addition of menthol (300μM), or ±PZQ (100μM), or (*R*)-PZQ or (*S*)-PZQ (50μM), followed by ACh (100μM). (**D**) Cumulative measurements of peak fluorescence ratio (F/F_0_, where ‘F’ represents fluorescence at peak and ‘F_0_’ represents fluorescence at time = 0) from Ca^2+^ imaging experiments under indicated conditions. AMTB (10μM) was added to cells 30min before addition of agonists. Data represent representing population mean±s.e.m. (≥20 cells) from n≥3 independent transfections. (**E**) Dose response relationship for (*S*)-PZQ evoked Ca^2+^ mobilization in TRPM8 expressing cells.

Given the efficacy of (*S*)-PZQ at causing vasorelaxation (Fig 2), the stereoselectivity of (*S*)-PZQ at hTRPM8 (Fig 3) and the proposed role for TRPM8 in mesenteric vascular beds [24, 25], secondary assays were performed using single cell confocal Ca^2+^ imaging to validate (*S*)-PZQ action at human TRPM8. Untransfected, and human TRPM8 transfected, HEK293 cells were challenged with ±PZQ, (*R*)-PZQ and (*S*)-PZQ and menthol (a TRPM8 agonist). In untransfected HEK293 cells, neither ±PZQ or menthol elevated cytoplasmic Ca^2+^ levels, while addition of acetylcholine (ACh) as a positive control caused Ca^2+^ transients through activation of endogenous muscarinic GPCRs (Fig 4A). However, in TRPM8 expressing cells, addition of menthol rapidly elevated cytoplasmic Ca^2+^ (Fig 4B), and this response was caused by Ca^2+^ entry as menthol-evoked Ca^2+^ signals were not observed in Ca^2+^-free media (Supplementary Fig 1). In TRPM8, expressing cells, addition of ±PZQ or (*S*)-PZQ evoked cytoplasmic Ca^2+^ signals, while (*R*)-PZQ was without effect (Fig 4B). Representative fluorescence traces for each of these experiments is shown in Fig 4C. Finally, Ca^2+^ transients evoked by either (*S*)-PZQ or menthol were blocked by the TRPM8 antagonist AMTB. The cumulative data for all the confocal Ca^2+^ imaging experiments is shown in Fig 4D. Analysis of the dose dependency of (*S*)-PZQ action on hTRPM8 revealed micromolar sensitivity (EC_50_ = 19.2±5.3 μM, Fig 4E). Collectively, these data validated the primary screen results evidencing stereoselective activation of TRPM8 by (*S*)-PZQ.

**Fig 5:**
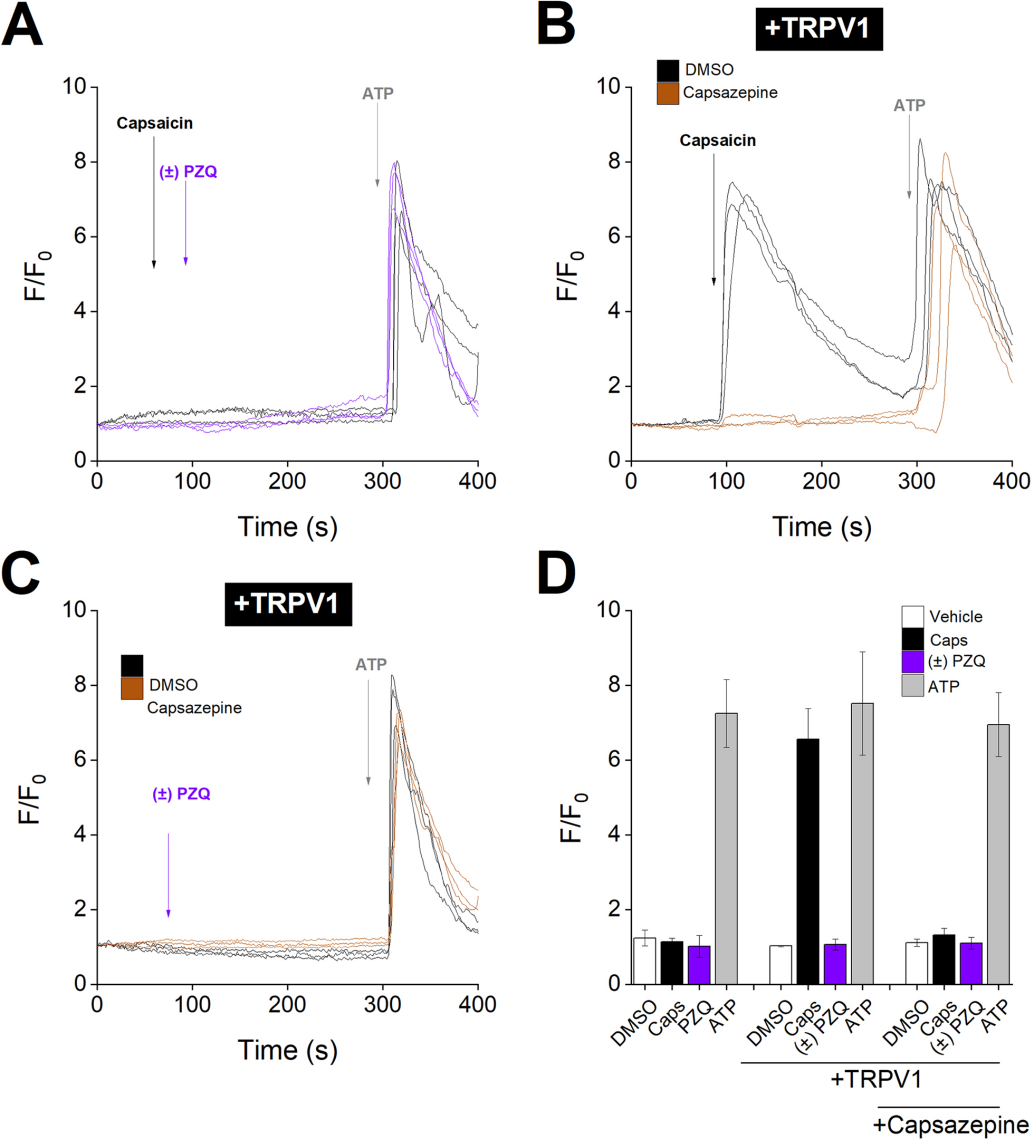
PZQ enantiomers do not activate heterologously expressed TRPV1. **(A)** Representative fluorescence traces from HEK293 cells loaded with Fluo-4 AM following the addition of capsaicin (1μM, black) or ±PZQ (100μM, purple), followed by addition of ATP (100μM). **(B)** Representative fluorescence traces from HEK293 cells transfected with hTRPV1 and loaded with Fluo-4 AM following the addition of capsaicin (1μM), followed by addition of ATP (100μM), in the presence of DMSO (0.1%, black) or capsazepine (10μM, brown). **(C)** Representative fluorescence traces of HEK293 cells transfected with hTRPV1 and loaded with Fluo-4 AM following the addition of ±PZQ (100μM), followed by ATP (100μM), in the presence of DMSO (0.1%, black), or capsazepine (10μM, brown). **(D)** Cumulative measurements of peak fluorescence ratio (F/F_0_, where ‘F’ represents fluorescence at peak and ‘F_0_’ represents fluorescence at time = 0) from Ca^2+^ imaging experiments under indicated conditions. Data represent population means±s.e.m. (>20 cells) from n≥3 independent transfections.

As a negative control for these experiments, we analyzed responses from human TRPV1-expressing cells: no activity of ±PZQ against TRPV1 was observed in the primary screen (Fig 3A). In untransfected HEK293 cells, neither the addition of the TRPV1 agonist capsaicin (1μM) nor addition of ±PZQ evoked a Ca^2+^ response (Fig 5A). However, in TRPV1 expressing cells, addition of capsaicin evoked Ca^2+^ signals which could be blocked by the TRPV1 antagonist, capsazepine (10μM, Fig 5B). No responses to ±PZQ (100μM) were observed under similar conditions (Fig 5C). The cumulative dataset from these assays is shown in Fig 5D. These data were consistent with the primary screen showing no activation of human TRPV1 by ±PZQ.

**Fig 6:**
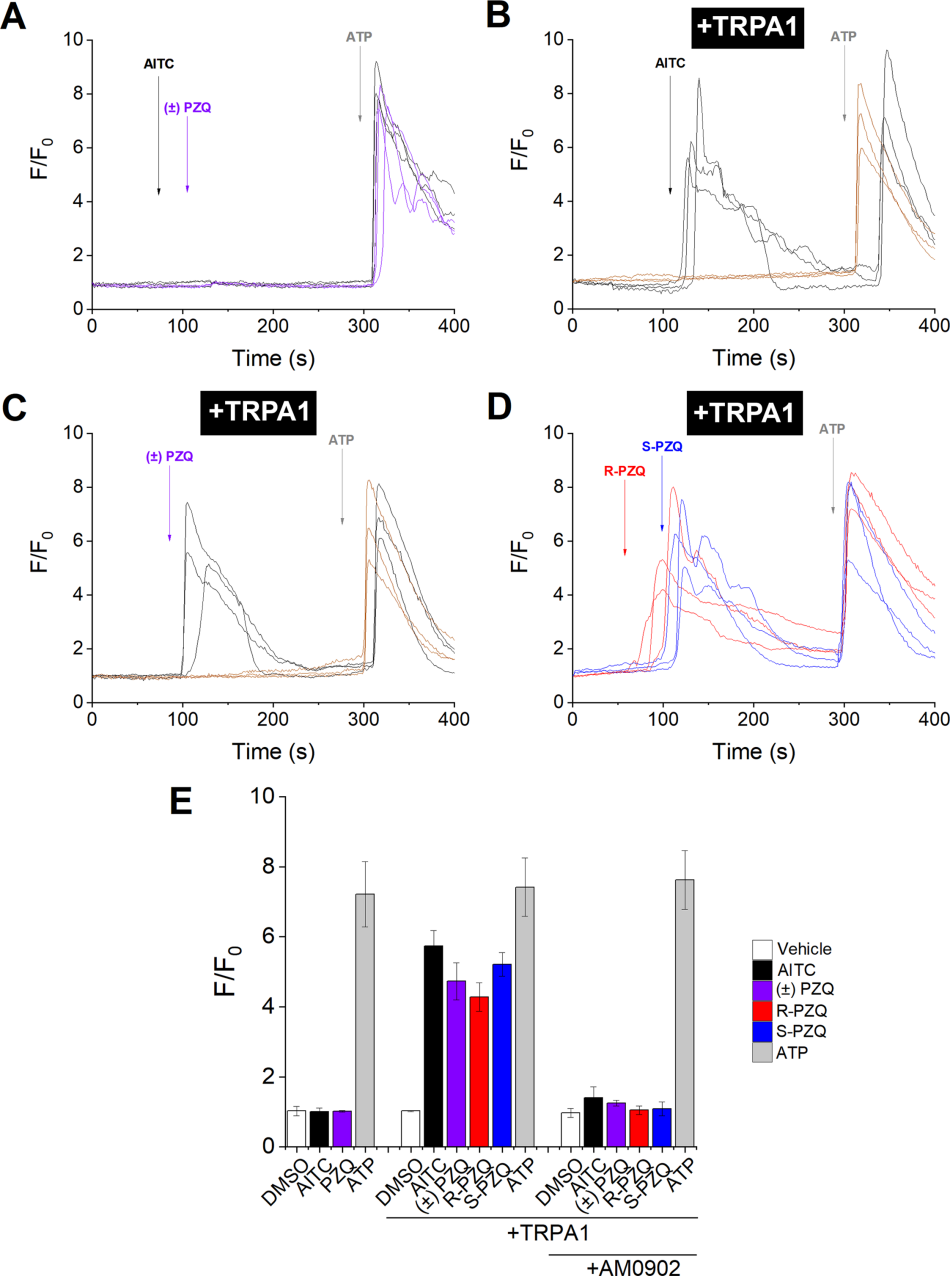
PZQ enantiomers activate TRPA1. **(A)** Representative fluorescence traces from HEK293 cells loaded with Fluo-4 AM following the addition of AITC (100μM, black) or ±PZQ (100μM, purple), followed by addition of ATP (100μM). **(B)** Representative fluorescence traces from HEK293 cells transfected with hTRPA1 and loaded with Fluo-4 AM following the addition of AITC (100μM), followed by addition of ATP (100μM), in the presence of DMSO (0.1%, black) or AM-0902 (1μM, brown). **(C)** Representative fluorescence traces of HEK293 cells transfected with hTRPA1 and loaded with Fluo-4 AM following the addition of ±PZQ (100μM), followed by ATP (100μM), in the presence of DMSO (0.1%, black), or AM-0902 (1μM, brown). **(D)** Representative fluorescence traces from HEK293 cells transfected with hTRPA1 and loaded with Fluo-4 AM following the addition of R-PZQ (100μM, red) or S-PZQ (100μM, blue), followed by addition of ATP (100μM). **(E)** Cumulative measurements of peak fluorescence ratio (F/F_0_, where ‘F’ represents fluorescence at peak and ‘F_0_’ represents fluorescence at time = 0) from Ca^2+^ imaging experiments under indicated conditions. Data represent representing population mean±s.e.m. (>20 cells) from n≥3.

Next, we performed secondary validation assays on TRPA1, shown to be activated by both PZQ enantiomers in the primary screen (Fig 3). In untransfected HEK293 cells, addition of ±PZQ (100μM), or the TRPA1 agonist allyl isothiocyanate (AITC, 100μM) was without effect, suggesting a lack of endogenously expressed TRPA1 channels (Fig 6A). However, in cells heterologously expressing hTRPA1, addition of AITC resulted in an elevation of cytoplasmic Ca^2+^, an effect which could be blocked by preincubation with the TRPA1 antagonist, AM-0902 (1μM), thus demonstrating functional expression of the hTRPA1 channel in transfected cells (Fig 6B). Addition of ±PZQ (100μM) to hTRPA1 expressing cells also elicited Ca^2+^ responses, which were also blocked by preincubation with AM-0902 (1μM) (Fig 6C). In contrast to hTRPM8, addition of either (*R*)-PZQ or (*S*)-PZQ resulted in activation of hTRPA1 (Fig 6D). Cumulative data for these TRPA1 assays in HEK293 cells are shown in Fig 6E. These data confirm the results of the primary screen showing activation of TRPA1 by (S)-PZQ and (R)-PZQ.

**Fig 7:**
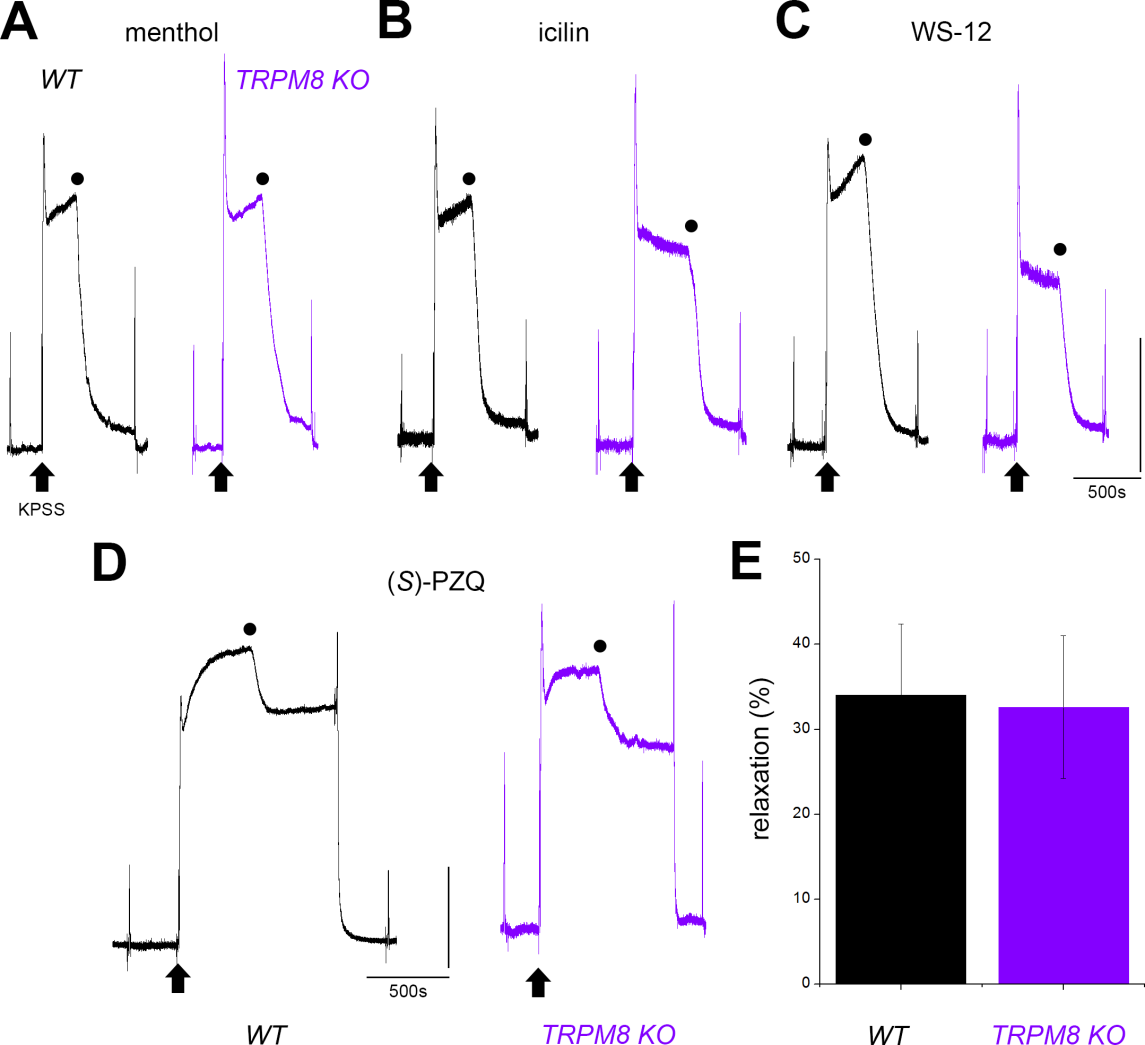
Comparison of responses to TRPM8 ligands in wild type and TRPM8 KO mice. (**A-C**) Responses to the TRPM8 agonists (A) menthol (300μM), (B) icilin (50μM) and (C) WS-12 (50μM) in wild type (WT, black) and TRPM8 knockout mice (TRPM8 KO, purple) when applied during the sustained phase of a KPSS-evoked contraction (shown by black circle). (**D**) Similar assay for (*S*)-PZQ (50μM) evoked relaxation during KPSS-evoked contraction in wild type (WT, black) and TRPM8 knockout mice (TRPM8 KO, purple). (**E**) Cumulative dataset measuring (*S*)-PZQ (50μM) evoked relaxation from experiments such as shown in (D). Data represent mean±s.e.m. from averaged measurements from mesenteric vessel strips from n≥3 mice.

Having established hTRPM8 as one target of (*S*)-PZQ (Figs 3&4), we returned to evaluate (*S*)-PZQ action within mesenteric blood vessels at endogenous levels of channel expression. First, various TRPM8 agonists were examined. These included menthol, icilin (a more potent small molecule structurally unrelated to menthol) and WS-12 (another potent menthol derivative). Each of these agents completely relaxed KPSS-contracted vessel strips at high concentrations (menthol 300μM, icilin 50 μM and WS-12 50μM, Fig 7A–7C). While suggestive of action at TRPM8, these compounds are known to display broader action within the TRP family, as well as affinity for other Ca^2+^ channels [26, 27]. Therefore, we repeated these experiments in mesenteric vessels isolated from a TRPM8 knockout mouse (TRPM8 KO). In the TRPM8 KO background, the vasorelaxant effect of the TRPM8 ligands persisted (Fig 7A–7C). The ability of (*S*)-PZQ to relax KPSS-evoked contractions was also examined in both models (Fig 7D), and the relaxant effect was preserved in TRPM8 KO tissue. The extent of relaxation (~30% of peak KPSS-evoked tone) was similar in WT and TRPM8 KO tissue (Fig 7E). These results indicate that TRPM8 does not mediate the vasorelaxation evoked by (*S*)-PZQ, and that the relaxation observed with TRPM8 agonists was caused by broader action against other targets. We therefore conclude that while (*S*)-PZQ is vasoactive over the micromolar range in mesenteric arteries, this effect is not mediated by TRPM8.

## Discussion

Here we demonstrate functional interactions between the resolved enantiomers of ±PZQ and a subset of human TRP channels over the micromolar range (Fig 3). These interactions may have significance for understanding the mechanism of action of ±PZQ in both host and parasite.

## Host target(s) of ±PZQ

In terms of host biology, this concentration range is compatible with (*R*)-PZQ and (*S*)-PZQ concentrations attained within the splanchnic vasculature during ±PZQ treatment [13, 28, 29]. While the majority of human TRP channels were unaffected by (*R*)-PZQ and (*S*)-PZQ (Fig 2), the subset of TRP channels engaged by PZQ enantiomers (hTRPA1, hTRPC3, hTRPC7, hTRPM8) are all expressed in host blood vessels inhabited by adult worm pairs, where their activation causes vasorelaxation. Activation of TRPC3 in mesenteric endothelium mediates agonist-evoked vasodilation [30–32], via various signaling mechanisms (nitric oxide (NO)-dependent signaling, hyperpolarization). TRPC7, which complexes with TRPC3 [33], mediates store-operated Ca^2+^ entry in portal vein myocytes [34]. TRPA1 activation also causes vasodilation: in mesenteric beds, this is mediated via TRPA1 activation releasing calcitonin gene related peptide (CGRP) from perivascular nerves. [35, 36]. Finally, TRPM8 is highly expressed in mesenteric artery and pharmacological activation of TRPM8 channels relaxes contracted vessels [20, 24, 25], effects attenuated in TRPM8 knockout mice [24]. These data suggest vasodilation of contracted blood vessels as a possible physiological outcome of host TRP channel engagement by ±PZQ. We note TRPM5, a transducer of bitter taste signaling was not activated by (*S*)-PZQ (Fig 3). While taste is a side effect associated with (*S*)-PZQ [14], another target in the bitter tasting pathway must explain this association.

The potential role for PZQ engagement of TRPs in vasodilatory responses was further bolstered by recent data showing that ±PZQ acts a partial agonist of TRPM8 over the micromolar range [15]. Expanding upon this discovery, we demonstrate here that ±PZQ activation of TRPM8 is mediated exclusively by the (*S*)-PZQ enantiomer (Figs 3&4), and given that (*S*)-PZQ is responsible for the vasodilatory effect observed in the myography experiments (Fig 2), these correlations prompted consideration of TRPM8 as the prime candidate for (*S*)-PZQ regulation *in vivo*.

However, analysis of vessel responses in TRPM8 KO tissue were inconsistent with this hypothesis, as vasorelaxation by either (*S*)-PZQ or TRPM8 agonists was unaffected by the loss of TRPM8 (Fig 6). Instead, vasodilation by TRPM8 ligands in response to K^+^-evoked depolarization likely reflects ‘off-target’ actions of these drugs. While this does not detract from evidence of host TRP regulation by the PZQ enantiomers (Figs 3&4), results from TRPM8 KO tissue do leave the molecular basis of the (S)-PZQ evoked vasorelaxation unresolved. One possibility is broader effects of ±PZQ on other PZQ-sensitive TRP channels (Fig 3) expressed in different cell types within the splanchnic circulation to coalesce vasodilatory cues on contracted vessels. Another possibility is that (S)-PZQ could be acting directly as a voltage-operated calcium channel blocker, consistent with data demonstrating non-specific blockade of voltage-operated Ca^2+^ channels (Ca_v_) by TRPM8 ligands in isolated arteries [26]. If indeed (S)-PZQ were to act as a Ca_v_ blocker, then the original Ca_v_ activation hypothesis of PZQ action [37–39] merits further attention. Could ±PZQ be acting in an analogous way to the Ca_v_ ligand ±BayK8644 [40], where one enantiomer acts as a Ca_v_ agonist ((*R*)-PZQ)—as implied previously [37–39], and one enantiomer ((*S*)-PZQ) as a Ca_v_ blocker—as implied here? Investigation of these possibilities is beyond the scope of the current study.

While conventionally viewed as a ‘selective’ antiparasitic therapy, our observations reinforce recent data demonstrating that the clinical racemate ±PZQ is vasoactive in the host [13]. Two discrete actions on host mesenteric vasculature are relevant, mediated by discrete enantiomers–first, constriction of basal tone ((*R*)-PZQ activation of 5-HT_2B_ receptors, [13]) and second, dilation of contracted vessels by (*S*)-PZQ. Both actions would occur on administration of ±PZQ, and could combine to optimize blood flow and perfusion pressure throughout the mesenteric vasculature to help flush (*R*)-PZQ paralyzed worms to the liver. Such changes in vascular tone may underpin the ‘hepatic shift’ seen *in vivo* on administration of either (*R*)-PZQ or (*S*)-PZQ [9] even though (*S*)-PZQ is lacks activity against adult schistosomes *in vitro*. The host targets of (*R*)-PZQ (5-HT_2B_) and (*S*)-PZQ (TRPM8) provide a rare example of enantiomers within a clinical formulation that target structurally distinct effectors (a GPCR versus a non-selective cation channel). The commonality between these targets may be realized by considering PZQ as a ‘tryptaminergic pharmacophore’, a view supported from studies of PZQ action in flatworms [41]. In addition to modulating serotoninergic binding pockets of GPCRs, tryptaminergic ligands also modulate the activity of TRPM8 [42], a channel notorious for activation by broad chemotypes. Tryptaminergic ligands of TRPM8 include 5-benzyloxytryptamine [43, 44], certain N-substituted tryptamines [45] and indole alkaloids [46].

Finally, in terms of interaction of PZQ enantiomers with human TRP channels, some comment on commonalities and discrepancies with prior results is warranted. Most importantly, our data confirm the key discovery of Babes *et al*. [15] that ±PZQ acts as a partial agonist at human TRPM8 over the micromolar range (±PZQ EC_50_ ~25μM by microfluorimetry [15], ±PZQ EC_50_ = 19±5μM by confocal imaging, Fig 4E). This activity is attributable to the (*S*)-enantiomer (Fig 4E). Other human TRP channels were also regulated by ±PZQ (Fig 3) and here our results contrast with prior data [15]. Babes *et al*. demonstrated a lack of activity of ±PZQ against TRPA1 (≤100μM), and show low potency activation of TRPV1 by ±PZQ (100μM). In contrast, our data show the opposite: ±PZQ activates TRPA1 (Figs 3&6), with no apparent activation of TRPV1 under our experimental conditions (Figs 3&5). The reason for these discrepancies is currently unclear but merits further investigation given the existence of homologs to TRPA1 in parasitic schistosomes, but not to TRPV1 and TRPM8 [18].

## Parasite target(s) of ±PZQ

Discovery of TRPs as human targets of ±PZQ is also informative for efforts to define the parasitic target(s) of ±PZQ, as precedent has now been established for ±PZQ action as both a GPCR ligand and TRP channel modulator. Despite the molecular divergence between human and flatworm proteins and ligand binding pockets [19, 47], it is not unreasonable to anticipate (*R*)-PZQ or (*S*)-PZQ affinities for flatworm target(s) within both the GPCR or TRP channel families. Both 5-HT_2B_R (G_q_ coupled) and the individual TRP channel targets (hTRPA1, hTRPC3, hTRPC7, hTRPM8) elevate cytoplasmic Ca^2+^, and the ability of ±PZQ to dysregulate Ca^2+^ homeostasis in both parasitic schistosomes and free-living flatworms is well appreciated [6, 48–50]. Moreover, the activity of serotonergic GPCRs and TRP channels can be coupled through amplifying interactions–GPCR mediated Ca^2+^ store depletion activates TRP mediated Ca^2+^ entry, which can itself stimulate serotoninergic pathways [51–53]. Perhaps the unique host-parasite polypharmacology of ±PZQ to engage reinforcing parasite targets deleterious to worm viability together with host pathways that mediate beneficial responses combating infection underpins the unique clinical efficacy of ±PZQ that has proved difficult to replicate over 35 years of clinical usage.

## Supporting information

S1 Fig Heterologously expressed TRPM8 mediates Ca^2+^ influx. Addition of menthol (300μM, first arrow) to TRPM8-expressing HEK293 cells does not cause a Ca^2+^ signal in Ca^2+^-free media, only when extracellular media is replaced with Ca^2+^-containing media. Traces represent fluorescence profiles from individual cells from a representative experiment.
(TIF)
